# COVID-19 pandemic and eating disorders: What impact on specific and general psychopathology?

**DOI:** 10.1192/j.eurpsy.2021.328

**Published:** 2021-08-13

**Authors:** E. Barone, F. Marciello, G. Cascino, G. Abbate-Daga, S. Anselmetti, M. Baiano, M. Balestrieri, S. Bertelli, B. Carpiniello, G. Castellini, G. Corrivetti, S. De Giorgi, A. Favaro, C. Gramaglia, E. Marzola, F. Monaco, M.G. Oriani, P. Federica, M. Rania, C. Renna, V. Ricca, P. Salvo, C. Segura-Garcia, F. Scarabel, P. Todisco, U. Volpe, P. Zeppegno, P. Monteleone, A.M. Monteleone

**Affiliations:** 1 Department Of Psychiatry, University of Campania “Luigi Vanvitelli”, Napoli, Italy; 2 Department Of Medicine, Surgery And Dentistry, University of Salerno, Baronissi/Salerno, Italy; 3 Department Of Neuroscience, Eating Disorders Center for Treatment and Research, torino, Italy; 4 Nutrimente Onlus, Nutrimente Onlus, Milan, Italy; 5 Department Of Psychiatry, Centro Unico Disturbi Comportamento Alimentare, udine, Italy; 6 University Of Udine, unit of psychiatry, DAME, udine, Italy; 7 Department Of Mental Health, ASST Santi Paolo e Carlo, Milano, Italy; 8 Italian Psychiatric Association; Secretary Of The Epa-council Of Npas, Department of Medical Sciences and Public Health University of Cagliari, Cagliari, Italy; 9 Department Of Health Science, University of Florence, Florence, Italy; 10 Department Of Psychiatry, 10European Biomedical Research Institute of Salerno (EBRIS), salerno, Italy; 11 Mental Health Department, Asl, Center for the Treatment and Research on Eating Disorders, lecce, Italy; 12 12department Of Neuroscience, University of Padova, padova, Italy; 13 Translational Medicine, Università Del Piemonte Orientale Amedeo Avogadro, Psychiatry Institute, Piermonte, Italy; 14 Department Of Mental Health, ASL Salerno, salerno, Italy; 15 Department Of Mental Health, ED outpatient service, jesi, Italy; 16 Department Of Health Science, university of Magna Grecia of Catanzaro, catanzaro, Italy; 17 Department Of Neuroscience, Psychology, Drug Research, Child Health, University of Florence, Florence, Italy; 18 Eating Disorders Centre Portogruaro, AULSS4 Veneto oientale, portogruaro, Italy; 19 Department Of Medical And Surgical Sciences, university of Magna Graecia of Catanzaro, catanzaro, Italy; 20 Eating Disorders Unit, Casa di cura “villa Margherita”, arcugnano, Italy; 21 Department Of Neurosciences/dimsc, School of Medicine Università Politecnica delle Marche, Ancona, Italy; 22 Department Of Medicine, Surgery And Dentistry “scuola Medica Salernitana”, University of Salerno, Baronissi/Salerno, Italy; 23 Department Of Psychiatry, University of Campania “Luigi Vanvitelli”, Naples, Italy

## Abstract

**Introduction:**

The coronavirus disease 2019 (COVID-19) pandemic and the resulting containment measures, such as “lockdown” and “social distancing”, have had important consequences on people’s mental and physical health.

**Objectives:**

We aimed to study the effect of social isolation and subsequent re- exposure and eventual changes in general and ED-specific psychopathology in people with Eating Disorders (EDs).

**Methods:**

Three-hundred twelve Italian people with EDs (179 Anorexia Nervosa, 83 Bulimia Nervosa, 48 Binge Eating Disorder and 22 Other Specific Feeding Eating Disorder) were asked to fill-in an online survey to explore several dimensions such as: anxiety, depression, panic, insomnia, suicide ideation, stress, post-traumatic stress and obsessive-compulsive symptoms. Differences in ED specific and general symptoms among the 3 investigated time periods (before, during and after the end of lockdown) were assessed with a one-way ANOVA with repeated measures. Subsequently, ED diagnosis was introduced as covariate in the analysis in order to investigate the possible contribution on psychopathological changes.

**Results:**

ED core symptoms increased during the lockdown but most of them returned to pre-COVID19 levels at re-opening. The severity of general psychopathology also increased during the lockdown and persisted high in the following phase, except for depression and suicide ideation. None of this symptoms was affected by ED diagnosis, participants’age and illness duration.

**Conclusions:**

People with EDs showed worsening of both general and specific psychopathology; moreover, changes in general psychopathology persisted in the re-opening period suggesting a higher stress vulnerability in this kind of patients.

**Disclosure:**

No significant relationships.

**Keyword:**

COVID19 and Eating Disorders

